# BinMat: A molecular genetics tool for processing binary data obtained from fragment analysis in R

**DOI:** 10.3897/BDJ.10.e77875

**Published:** 2022-03-11

**Authors:** Clarke van Steenderen

**Affiliations:** 1 Centre for Biological Control, Department of Zoology and Entomology, Rhodes University, Grahamstown/Makhanda, South Africa Centre for Biological Control, Department of Zoology and Entomology, Rhodes University Grahamstown/Makhanda South Africa

**Keywords:** AFLP, binary data scoring, GUI, ISSR, R package, R Shiny

## Abstract

Processing and visualising trends in the binary data (presence or absence of electropherogram peaks), obtained from fragment analysis methods in molecular biology, can be a time-consuming and often cumbersome process. Scoring and analysing binary data (from methods, such as AFLPs, ISSRs and RFLPs) entail complex workflows that require a high level of computational and bioinformatic skills. The application presented here (BinMat) is a free, open-source and user-friendly R Shiny programme (https://clarkevansteenderen.shinyapps.io/BINMAT/) that automates the analysis pipeline on one platform. It is also available as an R package on the Comprehensive R Archive Network (CRAN) (https://cran.r-project.org/web/packages/BinMat/index.html). BinMat consolidates replicate sample pairs of binary data into consensus reads, produces summary statistics and allows the user to visualise their data as ordination plots and clustering trees without having to use multiple programmes and input files or rely on previous programming experience.

## Introduction

Fragment analysis is a method in molecular biology that encompasses the processes by which fragments of DNA are separated by size in order to generate characteristic band profiles. Bands are detected and scored through either the traditional method of viewing them on polyacrylamide gels (Bassam et al. 1991) or through the use of fluorescent markers (such as FAM^TM^ or ROX) that tag fragments so that they can be detected by capillary electrophoresis (Dresler-Nurmi et al. 2000, Applied Biosystems 2014). There are a number of techniques associated with fragment analysis, including AFLP (Amplified Fragment Length Polymorphism) (Vos et al. 1995), RAPD (Random Amplified Polymorphic DNA) (Koeleman et al. 1998) and ISSR (Inter-Simple Sequence Repeats) (Wolfe and Liston 1998, Abbot 2001). Fragment analysis offers a wide range of applications, such as DNA fingerprinting, SNP (single nucleotide polymorphism) genotyping and microsatellite profiling (Applied Biosystems 2014), which are used across a broad range of disciplines.

Processing and analysing the binary data, obtained from fragment analysis methods, can quickly become challenging due to the large size of datasets and the time required to organise and format them to suit the needs of different programmes used in analysis pipelines. Common practice is to independently replicate each Polymerase Chain Reaction (PCR) sample in order to consolidate the output into one consensus read per individual (see, for example, Taylor et al. 2011 and Sutton et al. 2017). The term 'consolidate', as used here, refers to the process of checking the binary value scored at each locus position across every replicate pair and creating one representative consensus output for that sample. For example, if both replicates show the presence of a band at a particular locus, a '1' is recorded as 'present' at that locus. If a band was absent in both replicates, a '0' is recorded. If one replicate shows the presence of a band, but the other shows an absence, a '?' is recorded to denote an ambiguous read.

Manually consolidating the replicate pairs of large binary matrices in this way is not only impractical, but it also lends itself to human error. Even after fragments have been scored and processed, the downstream analyses of these data are complex. For example, a number of different programmes are often required for different analyses, each of which require a different input file format. This requires a certain level of computational and/or bioinformatic skills, can be both difficult and time-consuming and can result in further potential errors when changing between file formats.

The R programming language (RStudio Team 2020) is becoming an increasingly popular means of analysing genetic data (Paradis et al. 2004, Schliep 2011, Archer et al. 2017), as it can read in multiple file formats and perform a number of analyses all on one platform. Packages in R can, however, often be challenging to utilise for newcomers to programming. The development of a GUI (Graphical User Interface) can address this by collating multiple processing tools into one place and make complex computational tasks more accessible to researchers (see, for example, Reyes et al. 2019).

**Figure 1.**
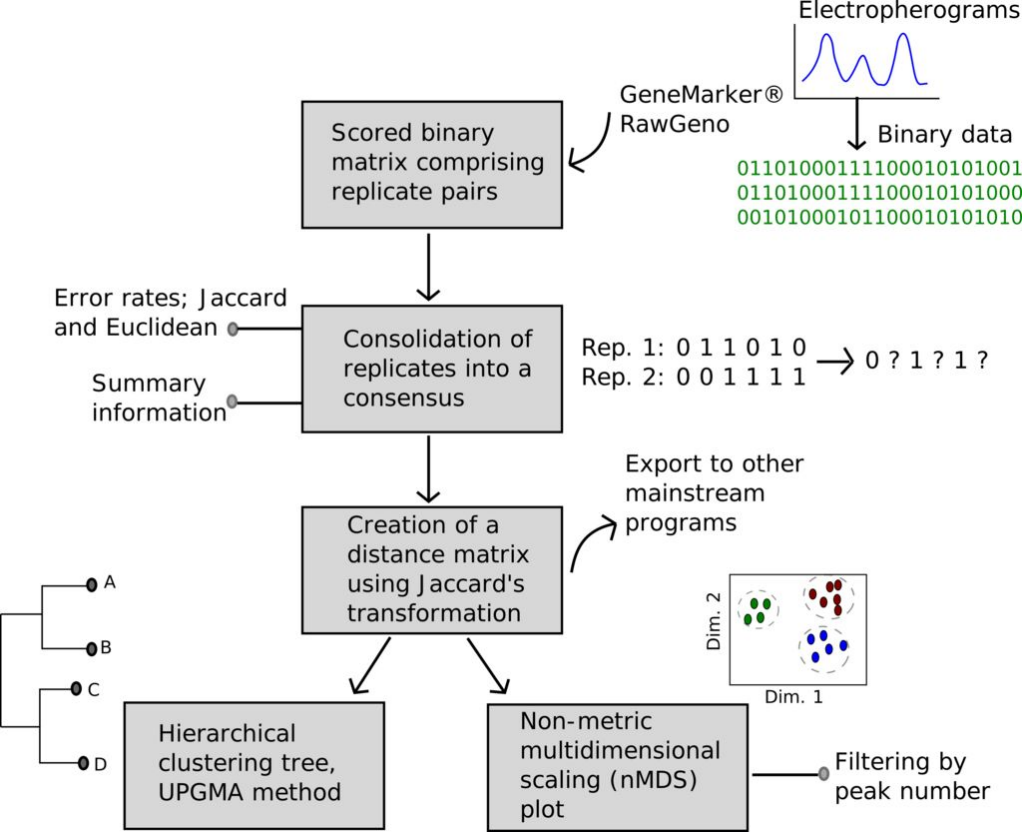
Flowchart of the utility of the BinMat programme, starting with input that has been processed in programmes such as GeneMarker and RawGeno, to the rapid visualisation of a hierarchical clustering tree and non-metric dimensional scaling (nMDS) plot.

Here, I present BinMat, an R package and R Shiny application that automates the analysis of fragment data. Named 'BinMat', from '**Bin**ary **Mat**rix', the application offers researchers a user-friendly, open-source platform that does not require multiple programmes and file input formats (Fig. 1). Moreover, a GUI was developed to make data processing easier and more accessible. BinMat is available on three platforms; namely the shinyapps.io server, GitHub and as an R package on CRAN. The following sections detail the functionality of BinMat, how its output compares to PAST (Hammer et al. 2001) and SplitsTree (Huson 1998) (which are standalone software typically used to analyse genetic data) and how it can be accessed.

## R Shiny graphical user interface

The R Shiny application platform allocates a maximum memory of 1 GB and is accessible here. The online version may time-out due to insufficient memory if a particularly large binary data file is uploaded. In such a case, the programme can be run directly from R on the user's local machine by typing

install.packages("shiny")

shiny::runGitHub("BinMat", "clarkevansteenderen")

into the console.

The programme's code is freely available on GitHub.

**File input**

BinMat reads in binary data that has already been processed from raw electropherograms using programmes such as GeneMarker (SoftGenetics) and RawGeno (Arrigo et al. 2012). This needs to be uploaded as a CSV (comma-separated values) file in the format shown in Table 1. Column headings are required, but are not limited to the exact labels shown in the example. If the data consist of replicate pairs, these need to be organised so that they appear consecutively, with a unique name for each sample. It is important to check the data to ensure that there are no single samples without their replicate. When the 'Consolidate matrix' button is clicked, each replicate pair in the dataset is consolidated into a consensus output.

Table 2 shows the output if the data in Table 1 were used as input. The resulting consolidated binary matrix can be downloaded as a CSV file using the 'Download Matrix' button once the message 'COMPLETE. READY FOR DOWNLOAD' appears on the screen. The 'Check my data for unwanted values' button checks the data for any values in the dataset other than a '1', '0', or '?' and returns the column and row index for the unwanted character/s.

**Output overview**

Once the data have been consolidated, the user can view and download information in the 'SUMMARY' tab at the top of the window; showing the average number of peaks (± standard deviation (sd)), the maximum and minimum number of peaks and the total number of loci. The 'ERROR RATES' tab shows the Euclidean (EE) (± sd) and Jaccard (JE) (± sd) error rates. See Bonin et al. 2004, Pompanon et al. 2005 and Holland et al. 2008 for detailed reviews regarding error rates and their calculation.

The 'Remove samples with a jaccard error greater than' button removes samples with a Jaccard error (ranging from 0 to 1) greater than or equal to a specified value. This can give the user an idea of how filtering their data can affect overall error rates. The default value is set at zero.

Clustering methods, such as the UPGMA (Unweighted Pair Group Method with Arithmetic Mean) and neighbour-joining, are frequently used in the analyses of fragment data to create dendrograms (e.g. Van Eldere et al. 1999, Ticknor et al. 2001, Liu et al. 2009, Timm et al. 2010). Additionally, ordination methods, such as those offered by non-metric multidimensional scaling (nMDS) plots, are also often used (see, for example, Denaro et al. 2005, Zhang et al. 2008, Vašek et al. 2017).

**Hierarchical clustering tree: UPGMA**

The 'UPGMA TREE' tab in BinMat allows the user to upload a consolidated binary matrix as a CSV file (in the format shown in Table 2), specify the number of bootstrap replications and download the resulting hierarchical clustering tree as a scalable vector graphics (SVG) file. This function makes use of the pvclust function in the pvclust package (Suzuki and Shimodaira 2006) and uses the UPGMA clustering method. The uploaded binary data are converted into a distance matrix applying the Jaccard transformation (dJ_i_) (Jaccard 1908) shown below. **f_11_** represents the total number of times that a band occurred at the same locus in both samples, **f_00_** represents the shared absence of bands and **f_10_** and **f_01_** represents the number of times that a band was present in only one of the two sample replicates. The Jaccard transformation was applied using the *dist* function, applying the 'binary' method. This transformation was prefered because it does not treat the shared absence of bands as being biologically meaningful.

\begin{varwidth}{50in}
        \begin{equation*}
            dJi = \frac{f01 + f10}{f01 + f10 + f11}
        \end{equation*}
    \end{varwidth}

**Ordination: nMDS Plot**

The 'nMDS PLOT' tab allows the user to upload a consolidated binary matrix with grouping information as a CSV file. The input file format is shown in Table 3, where grouping information needs to appear in the second column.

The distance methods available are 'binary' (Jaccard's distance), 'euclidean', 'maximum', 'manhattan', 'canberra' and 'minkowski'. The 'No. of dimensions (k)' option can be set at '2' or '3' and can be determined using the 'nMDS Validation' tab using the 'Scree plot' and 'Shepard plot' buttons. The resulting distance matrix can be downloaded as a CSV file and the plot itself as a SVG file. Once the user has uploaded their data, an editable table will appear to allow for the selection of colours and symbols for each group. The user can adjust symbol size and can select whether sample labels should appear on the graph or not. The nMDS plot is created using the isoMDS function in the MASS package (Venables and & Ripley 2002).

**Scree plot**

The optimal number of dimensions to use for the nMDS plot should minimise the resulting stress value. Clarke 1993 suggests that stress values < 0.05 = excellent, < 0.10 = good, < 0.20 = usable, > 0.20 = not acceptable, while Dugard et al. 2010 suggest that a stress value below 0.15 represents a good fit for the data. BinMat indicates the 0.15 threshold as a dotted red line on the resulting scree plot.

**Shepard plot**

Shepard plots are graphical representations of how well the ordination fits the original distance data (Leeuw and Mair 2014). BinMat plots the original Jaccard distances (x-axis) against the transformed distances used to create the nMDS ordination plot (y-axis). R^2^ values are shown on the plot for the regression line of best fit.

**Filter data**

The 'Filter data' tab allows the user to filter their dataset by setting a threshold value for the number of peaks present. The new subsetted data and the removed samples can be downloaded as a CSV file and re-uploaded to create a new nMDS plot and/or hierarchical clustering tree.

## Testing BinMat

**Comparing BinMat's output to PAST and SplitsTree**

**Figure 2.**
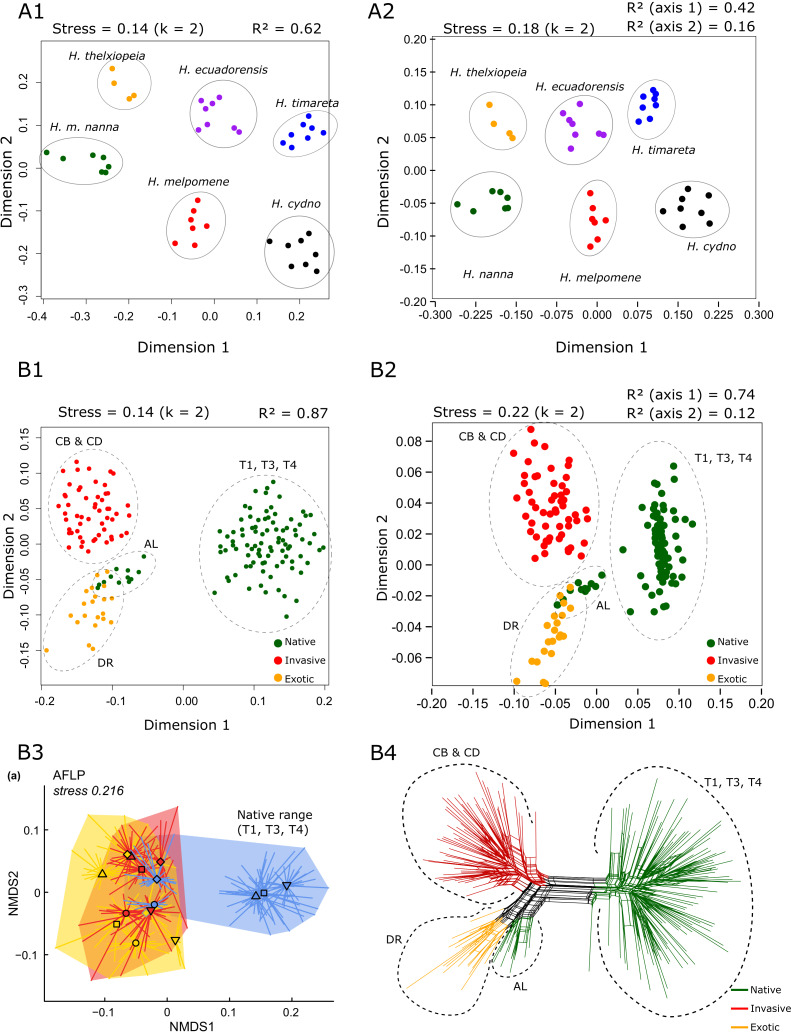
Comparisons of non-metric multidimensional scaling (nMDS) plots in BinMat (**A1** and **B1**) and PAST (**A2** and **B2**). Both nMDS plots are plotted for k = 2 dimensions. Data were taken from Arias et al. 2014 (A1 and A2) and Tewes et al. 2018 (**B1**, **B2** and **B4**). Stress and R^2^ values are shown above each plot. Diagram B3 shows the original nMDS plot presented by Tewes et al. 2018, which depicts the same clustering pattern of the native range samples (T1, T3 and T4). Diagram B4 shows the SplitsTree representation of the same data (NeighborNet, Jaccard distance).

Two AFLP datasets were downloaded from the Dryad Digital Repository. These comprised data generated by Arias et al. 2014 and Tewes et al. 2018 for *Heliconius* (Lepidoptera: Nymphalidae) and *Bunias orientalis* L. (Brassicaceae) specimens, respectively. With the authors' permission, a subset of each were used to compare output from BinMat to that of PAST v.4.0 (Paleontological Statistics Software Package for Education and Data Analysis) (Hammer et al. 2001) and SplitsTree v.4.14.6 (Huson 1998) (input data are available in Suppl. materials 1, 2, 3, 4, 5, 6, 7). Replicate pairs were consolidated in BinMat and used to create nMDS plots and UPGMA hierarchical clustering trees (1000 bootstrap repetitions). The lowest number of dimensions were used for nMDS plots (k = 2) and their stress and R^2^ values recorded. SplitsTree was used to create a NeighborNet tree applying Jaccard's distance transformation. The nMDS plots created by BinMat and PAST showed comparable clustering patterns (Fig. 2A1, A2, B1 and B2).

**Figure 3.**
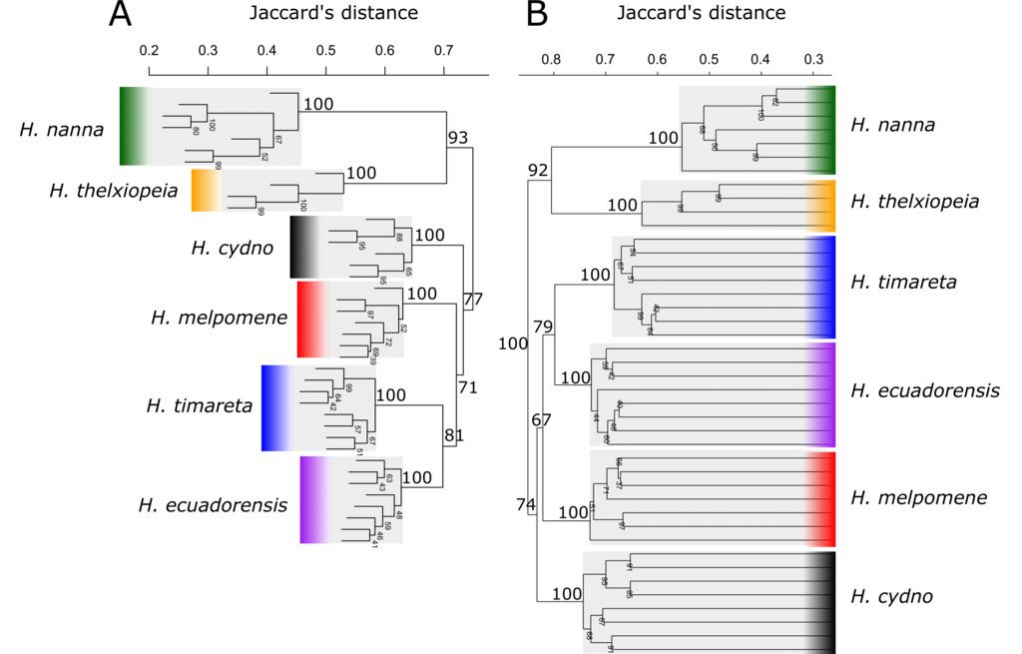
Comparison of hierarchical clustering trees in A) BinMat and B) PAST using the data taken from Tewes et al. 2018. Both programmes applied Jaccard's transformation to create a distance matrix and used the UPGMA clustering method. Bootstrap probabilities are shown on the branches, resulting from 1000 bootstrap repetitions.

The SplitsTree output for the data taken from Tewes et al. 2018 (Fig. 2B4) corroborated the corresponding nMDS plot from the original paper (Fig. 2B3) and from that created by BinMat (Fig. 2B1). Both hierarchical clustering trees using the UPGMA method showed equivalent topologies and bootstrap support values for clades (Fig. 3). BinMat, PAST and SplitsTree perform equally as well for the visualisation of fragment analysis output, where BinMat offers the advantage of a quicker, automated process on one platform.

## BinMat as an R package on CRAN

The BinMat R package is available on the Comprehensive R Archive Network (CRAN) and on GitHub and is command-line driven. More information about the package can be obtained by typing

library(help = BinMat)

into the console after it has been installed. This details all the functions available (Table 4).

To cite BinMat, use

citation("BinMat")

There are four example binary matrices embedded in the BinMat package called "BinMatInput_reps", "BinMatInput_ordination", "bunias_orientalis" and "nymphaea" that can be accessed by creating objects such as:

data1 = BinmatInput_reps

data2 = BinmatInput_ordination

These binary matrices can be used to test the various functions as a demonstration example, as shown in the worked example in the vignette supplied with the package. The "BinMatInput_reps" and "BinMatInput_ordination" are small hypothetical datasets, illustrating how BinMat consolidates replicate pairs and then creates an nMDS plot coloured by groups (e.g. populations). The "bunias_orientalis" and "nymphaea" datasets are real-world AFLP and ISSR results from Tewes et al. 2018 and Reid et al. 2021, respectively. These two datasets have already been consolidated and serve as examples for the generation of nMDS plots.

## Conclusion

BinMat offers users of fragment analysis methods an efficient and easy-to-use platform to process their binary data matrices, by means of either a graphical user interface or an R package. The programme produces comparable output to other mainstream software, with the benefit of housing all of its functionality on one platform. Suggestions for improvement (for example via pull-requests on GitHub) and feedback from the community, are welcomed.

## Supplementary Material

BF8F4046-06EC-5BCC-A4FD-8F443AE43A23 10.3897/BDJ.10.e77875.suppl1 Supplementary material 1 Arias et al. (2014) consolidated binary matrix with groups Data type Binary data (AFLP) Brief description Consolidated AFLP binary data from Arias et al. (2014), with a grouping column. This is used as input to BinMat for the creation of an nMDS plot. File: oo_607200.csv https://binary.pensoft.net/file/607200 Adapted from Arias et al. (2014)

FA23B93F-784F-5692-9BF6-7C9133B9445F 10.3897/BDJ.10.e77875.suppl2 Supplementary material 2 Arias et al. (2014) consolidated binary matrix without groups Data type AFLP binary data Brief description Consolidated AFLP binary data from Arias et. al. (2014), without a grouping column. File: oo_607202.csv https://binary.pensoft.net/file/607202 Adapted from Arias et al. (2014)

E6C96427-5F45-5577-B505-A11636E67933 10.3897/BDJ.10.e77875.suppl3 Supplementary material 3 Arias et al. (2014) raw AFLP binary data Data type AFLP binary data Brief description Raw AFLP binary data from Arias et al. (2014), before replicates have been consolidated. File: oo_607203.csv https://binary.pensoft.net/file/607203 Adapted from Arias et al. (2014)

14BD9736-C6B0-5884-B168-ADD70F000D78 10.3897/BDJ.10.e77875.suppl4 Supplementary material 4 Tewes et al. (2018) AFLP binary data for native and invasive Bruniasorientalis species Data type AFLP binary data in NEXUS format Brief description A NEXUS file containing AFLP binary data for native and invasive *Bruniasorientalis* species from the Tewes et al. (2018) study. This file is used as input for the SplitsTree programme. File: oo_607173.nex https://binary.pensoft.net/file/607173 Adapted from Tewes et al. (2018)

68166370-ED72-5C35-A80E-E79AF6F71124 10.3897/BDJ.10.e77875.suppl5 Supplementary material 5 Tewes et al. (2018) consolidated binary matrix with groups Data type Consolidated AFLP binary data Brief description Consolidated AFLP binary data from Tewes et. al. (2018), with a grouping column. This is used as input to BinMat for the creation of an nMDS plot. File: oo_607204.csv https://binary.pensoft.net/file/607204 Adapted from Tewes et al. (2018)

EE6E9EFA-33BE-52CA-B41A-D748904C9517 10.3897/BDJ.10.e77875.suppl6 Supplementary material 6 Tewes et al. (2018) consolidated binary matrix without groups Data type Consolidated AFLP binary data Brief description Tewes et al. (2018) consolidated AFLP binary data without a grouping column. File: oo_607205.csv https://binary.pensoft.net/file/607205 Adapted from Tewes et al. (2018)

83FA2DC5-5AB2-54B1-9116-EDF5922EB434 10.3897/BDJ.10.e77875.suppl7 Supplementary material 7 Tewes et al. (2018) raw binary AFLP data Data type Binary AFLP data Brief description Raw AFLP binary data from Tewes et al. (2018), before replicates have been consolidated. File: oo_607206.csv https://binary.pensoft.net/file/607206 Adapted from Tewes et al. (2018)

## Figures and Tables

**Table 1.** File input for a dataset containing replicate pairs that needs to be consolidated.

**Sample label**	**Locus 1**	**Locus 2**	**Locus 3**	**Locus 4**	**Locus 5**
Sample A rep 1	0	0	1	1	1
Sample A rep 2	0	0	1	1	1
Sample B rep 1	1	1	0	0	0
Sample B rep 2	0	1	0	0	1

**Table 2.** A consolidated matrix derived from Table 1 using BinMat.

**Sample label**	**Locus 1**	**Locus 2**	**Locus 3**	**Locus 4**	**Locus 5**
Sample A rep 1 + Sample A rep 2	0	0	1	1	1
Sample B rep 1 + Sample B rep 2	?	1	0	0	?

**Table 3.** Data input required for the creation of a non-metric multidimensional scaling (nMDS) plot. Grouping information needs to be in the second column. The data here represents binary replicate pairs that have already been consolidated into consensus reads.

**Sample label**	**Group**	**Locus 1**	**Locus 2**	**Locus 3**	**Locus 4**	**Locus 5**
Sample A	Africa	0	0	1	1	1
Sample A	Asia	?	1	0	0	?

**Table 4.** BinMat R package functions available on CRAN. Typing ?**functionName** into the console provides more information about each function.

**Function**	**Description**
check.data()	Checks for unwanted characters.
consolidate()	Consolidates replicate pairs. 1 & 1 = 1; 1 & 0 = ?; 0 & 0 = 0
errors()	Calculates Jaccard and Euclidean error rates.
group.names()	Outputs groups in the uploaded binary matrix.
nmds()	Creates a non-metric multidimensional scaling (nMDS) plot.
peak.remove()	Removes samples with peaks equal to, or less than, a specified threshold value.
peaks.consolidated()	Peak summary for a consolidated binary matrix.
peaks.orignal()	Peak summary for replicate data or consolidated data from file.
scree()	Creates a scree plot of stress values vs. ordination dimensions.
shepard()	Creates a shepard plot for goodness-of-fit for ordination data.
upgma()	Draws a hierarchical clustering tree (UPGMA) with bootstrapping.

## Hosting institution

Centre for Biological Control, Department of Zoology and Entomology, Rhodes University, South Africa

## References

[B7542979] Abbot Patrick (2001). Individual and population variation in invertebrates revealed by Inter-simple Sequence Repeats (ISSRs). Journal of Insect Science.

[B7542939] Biosystems Applied (2014). DNA fragment analysis by capillary electrophoresis.

[B7542886] Archer Frederick I, Adams Paula E, Schneiders Brita B (2017). stratag: An R package for manipulating, summarizing and analysing population genetic data. Molecular Ecology Resources.

[B7542781] Arias Carlos F, Salazar Camilo, Rosales Claudia, Kronforst Marcus R, Linares Mauricio, Bermingham Eldredge, McMillan W Owen (2014). Phylogeography of *Heliconiuscydno* and its closest relatives: disentangling their origin and diversification. Molecular Ecology.

[B7542844] Arrigo Nils, Holderegger Rolf, Alvarez Nadir, Pompanon F., Bonin A. (2012). Data production and analysis in population genomics.

[B7543021] Bassam Brant J, Caetano-Anollés Gustavo, Gresshoff Peter M (1991). Fast and sensitive silver staining of DNA in polyacrylamide gels. Analytical Biochemistry.

[B7542857] Bonin A, Bellemain E, Bronken Eidesen P, Pompanon F, Brochmann C, Taberlet P (2004). How to track and assess genotyping errors in population genetics studies. Molecular Ecology.

[B7542772] Clarke K Robert (1993). Non-parametric multivariate analyses of changes in community structure. Australian Journal of Ecology.

[B7543072] Denaro R, D’auria G, Di Marco G, Genovese M, Troussellier M, Yakimov MM, Giuliano L (2005). Assessing terminal restriction fragment length polymorphism suitability for the description of bacterial community structure and dynamics in hydrocarbon-polluted marine environments. Environmental Microbiology.

[B7543001] Dresler-Nurmi Aneta, Terefework Zewdu, Kaijalainen Seppo, Lindström Kristina, Hatakka Annele (2000). Silver stained polyacrylamide gels and fluorescence-based automated capillary electrophoresis for detection of amplified fragment length polymorphism patterns obtained from white-rot fungi in the genus *Trametes*. Journal of Microbiological Methods.

[B7542802] Dugard P, Todman J, Staines H (2010). Approaching multivariate analysis. A practical introduction.

[B7542793] Hammer Oyvind, Harper David A T, Ryan Paul D (2001). PAST: Paleontological statistics software package for education and data analysis. Palaeontologia Electronica.

[B7542877] Holland Barbara R, Clarke Andrew C, Meudt Heidi M (2008). Optimizing automated AFLP scoring parameters to improve phylogenetic resolution. Systematic Biology.

[B7543113] Huson Daniel H (1998). SplitsTree: analyzing and visualizing evolutionary data. Bioinformatics (Oxford, England).

[B7542913] Jaccard Paul (1908). Nouvelles recherches sur la distribution florale. Bulletin de la Societe Vaudoise des Sciences Naturelles.

[B7543011] Koeleman Johannes GM, Stoof Jeroen, Biesmans Dennis J, Savelkoul Paul HM, Vandenbroucke-Grauls Christina MJE (1998). Comparison of amplified ribosomal DNA restriction analysis, random amplified polymorphic DNA analysis, and amplified fragment length polymorphism fingerprinting for identification of *Acinetobacter* genomic species and typing of *Acinetobacterbaumannii*. Journal of Clinical Microbiology.

[B7543104] Leeuw Jan De, Mair Patrick (2014). Shepard diagram. Wiley StatsRef: Statistics Reference Online.

[B7543040] Liu Li-jun, Meng Zu-Qing, Wang Bo, Wang Xu-xia, Yang Jin-Yu, Peng Ding-xiang (2009). Genetic diversity among wild resources of the genus *Boehmeria* Jacq. from west China determined using inter-simple sequence repeat and rapid amplification of polymorphic DNA markers. Plant Production Science.

[B7542895] Paradis Emmanuel, Claude Julien, Strimmer Korbinian (2004). APE: analyses of phylogenetics and evolution in R language. Bioinformatics.

[B7542868] Pompanon François, Bonin Aurélie, Bellemain Eva, Taberlet Pierre (2005). Genotyping errors: causes, consequences and solutions. Nature Reviews Genetics.

[B7718140] Reid Megan K., Naidu Prinavin, Paterson Iain D., Mangan Rosie, Coetzee Julie A. (2021). Population genetics of invasive and native Nymphaea mexicana Zuccarini: Taking the first steps to initiate a biological control programme in South Africa. Aquatic Botany.

[B7542963] Reyes Alberto Luiz P, Silva Tiago C, Coetzee Simon G, Plummer Jasmine T, Davis Brian D, Chen Stephanie, Hazelett Dennis J, Lawrenson Kate, Berman Benjamin P, Gayther Simon A (2019). GENAVi: a shiny web application for gene expression normalization, analysis and visualization. BMC Genomics.

[B7542836] Team RStudio (2020). RStudio: Integrated development for R.

[B7542904] Schliep Klaus Peter (2011). Phangorn: Phylogenetic analysis in R. Bioinformatics.

[B7542819] Sutton G F, Paterson I D, Paynter Q (2017). Genetic matching of invasive populations of the African tulip tree, *SpathodeaCampanulata* Beauv. (Bignoniaceae), to their native distribution: Maximising the likelihood of selecting host-compatible biological control agents. Biological Control.

[B7549333] Suzuki Ryota, Shimodaira Hidetoshi (2006). Pvclust: an R package for assessing the uncertainty in hierarchical clustering. Bioinformatics.

[B7542810] Taylor S J, Downie D A, Paterson I D (2011). Genetic diversity of introduced populations of the water hyacinth biological control agent *Eccritotarsuscatarinensis* (Hemiptera: Miridae). Biological Control.

[B7718150] Tewes Lisa Johanna, Michling Florian, Koch Marcus A., Müller Caroline (2018). Intracontinental plant invader shows matching genetic and chemical profiles and might benefit from high defence variation within populations. Journal of Ecology.

[B7543060] Ticknor Lawrence O, Kolstø Anne-Brit, Hill Karen K, Keim Paul, Laker Miriam T, Tonks Melinda, Jackson Paul J (2001). Fluorescent amplified fragment length polymorphism analysis of Norwegian *Bacilluscereus* and *Bacillusthuringiensis* soil isolates. Applied and Environmental Microbiology.

[B7543051] Timm AE, Geertsema H, Warnich L (2010). Population genetic structure of economically important Tortricidae (Lepidoptera) in South Africa: a comparative analysis. Bulletin of Entomological Research.

[B7543030] Van Eldere Johan, Janssen Paul, Hoefnagels-Schuermans Annette, van Lierde Stefaan, Peetermans Willy E (1999). Amplified- fragment length polymorphism analysis versus macro-restriction fragment analysis for molecular typing of *Streptococcuspneumoniae* isolates. Journal of Clinical Microbiology.

[B7543093] Vašek Jakub, Čepková Petra Hlásná, Viehmannová Iva, Ocelak Martin, Huansi Danter Cachique, Vejl Pavel (2017). Dealing with AFLP genotyping errors to reveal genetic structure in *Plukenetiavolubilis* (Euphorbiaceae) in the Peruvian Amazon. PLOS One.

[B7681179] Venables W. N., & Ripley B. D. (2002). Modern Applied Statistics with S.

[B7542947] Vos Pieter, Hogers Rene, Bleeker Marjo, Reijans Martin, Lee Theo van de, Hornes Miranda, Friters Adrie, Pot Jerina, Paleman Johan, Kuiper Martin (1995). AFLP: a new technique for DNA fingerprinting. Nucleic Acids Research.

[B7542988] Wolfe Andrea D, Liston Aaron, Soltis D., Soltis P., Doyle J. (1998). Molecular Systematics of Plants II.

[B7543084] Zhang Rui, Thiyagarajan Vengatesen, Qian Pei-Yuan (2008). Evaluation of terminal-restriction fragment length polymorphism analysis in contrasting marine environments. Federation of European Microbiological Societies (FEMS) Microbiology Ecology.

